# Cerebral Endothelial Function Determined by Cerebrovascular Reactivity to L-Arginine

**DOI:** 10.1155/2014/601515

**Published:** 2014-04-17

**Authors:** Janja Pretnar-Oblak

**Affiliations:** Department for Vascular Neurology, Ljubljana Medical Centre, Zaloska 2, 1000 Ljubljana, Slovenia

## Abstract

Endothelium forms the inner cellular lining of blood vessels and plays an important role in many physiological functions including the control of vasomotor tone. Cerebral endothelium is probably one of the most specific types but until recently it was impossible to determine its function. In this review, the role of cerebrovascular reactivity to L-arginine (CVR-L-Arg) for assessment of cerebral endothelial function is discussed. L-Arginine induces vasodilatation through enhanced production of nitric oxide (NO) in the cerebral endothelium. Transcranial Doppler sonography is used for evaluation of cerebral blood flow changes. The method is noninvasive, inexpensive, and enables reproducible measurements. CVR-L-Arg has been compared to flow-mediated dilatation as a gold standard for systemic endothelial function and intima-media thickness as a marker for morphological changes. However, it seems to show specific cerebral endothelial function. So far CVR-L-Arg has been used to study cerebral endothelial function in many pathological conditions such as stroke, migraine, etc. In addition CVR-L-Arg has also proven its usefulness in order to show potential improvement after pharmacological interventions. In conclusion CVR-L-Arg is a promising noninvasive research method that could provide means for evaluation of cerebral endothelial function in physiological and pathological conditions.

## 1. Introduction

The endothelium forms the inner cellular lining of blood vessels. Endothelial cells are highly metabolically active and play an important role in many physiological functions, including the control of vasomotor tone, blood cell trafficking, haemostatic balance, permeability, proliferation, and immunity. Endothelial function is not uniform throughout the arterial system. It differs between organs and potentially also between different vascular beds within the same organ [1]. Endothelial cell phenotypes are differentially regulated in space and time, giving rise to the phenomenon of endothelial cell heterogeneity. Cerebral endothelium is probably one of the most specific types since it is the crucial element of the well-known blood-brain barrier. Cerebral endothelial dysfunction is mentioned in the pathophysiology of several neurological diseases. Until recently it was impossible to determine specific cerebral endothelial function.

In the past few decades the immense development of neuroradiological methods enabled better imaging of cerebral blood vessels. Noninvasive imaging of asymptomatic brain aneurysms, arteriovenous malformations, and intracranial arterial stenosis became available. In addition, imaging of the cerebral function became possible by functional magnetic resonance imaging (fMRI) and positron emission tomography (PET) scan. Although the bones of the scull remain an obstacle for ultrasound waves and therefore ultrasound may not seem to be the optimal technique for cerebral vessel imaging, transcranial ultrasound techniques were improved and found their place and indications. Ultrasound remains the ultimate method for real time functional cerebral blood flow imaging. It is unique in being able to detect blood flow velocity and direction of flow. Different techniques developed but the original transcranial Doppler sonography (TCD) still has several advantages. TCD enables continuous monitoring of mean blood flow velocity through the cerebral arteries and therefore the evaluation of cerebral blood flow [2].

Cerebral endothelium is a one cell layer on the inner wall of the vessels and as such is a difficult subject to image. Intraluminal ultrasound techniques enable imaging of morphological changes. However, these techniques do not enable any evaluation of cerebral endothelial function. In the past few years cerebrovascular reactivity to L-arginine by means of TCD has emerged as a parameter for evaluation of cerebral endothelial function [3–7].

### 1.1. Cerebral Blood Flow and the Role of Endothelium

Cerebral autoregulation maintains constant blood flow (CBF) through the brain in spite of changing mean arterial pressure [8]. Autoregulation of cerebral blood flow consists of mechano- and chemoregulation. Chemoregulation is in direct correlation to the serum level of carbon dioxide (CO_2_) [8] and is, contrary to mechanoregulation, independent of changes in mean arterial pressure. Mechanoregulation depends on transmural pressure gradient and endothelial vasodilatation. Mechanoregulation has been shown to be the main supervisory mechanism of CBF [9]. However, it is well established that endothelial vasodilatation of greater arteries is much more pronounced in cerebral vasculature than elsewhere.

Proper endothelial function is of crucial importance in regulation in many vascular beds [10]. Dysfunctional cerebral endothelium releases less endothelial NO. As a consequence relaxation of smooth muscle cells of small arteries is disturbed. Studies in animals and humans have revealed that mechanoregulation is not compromised even in older age and pathological conditions harming endothelium [11, 12]. On the contrary, outcomes of many studies and clinical reports confirm the dependency of chemoregulation of CBF on vascular endothelial integrity [13]. Reduced chemoregulation was found in patients with dysfunctional cerebral endothelium [14].

The importance of endothelial vasomotor role has been established during the last few decades. Mediators of vasodilatation have been studied, acting through endothelial G-protein coupled receptors (acetylcholine, bradykinin, and ATP). Intermediate mediators have been proposed, such as NO, some prostanoids, and endothelially derived hyperpolarization factor (EDHF). Vasoconstrictive mediators are also released by endothelium, for example, endothelin-1. Having in mind the complexity beyond the endothelial vasomotor activity, one can conclude that any derangement of endothelium may lead to its dysfunction. Preserved endothelial function is of utmost importance in regulating cerebral blood circulation.

The majority of the above conclusions are based on animal studies. However, several techniques enable evaluation of cerebral blood flow in humans. They have their advantages and disadvantages. Methods such as single photon emission computed tomography (SPECT), positron emission tomography (PET), and stable xenon-enhanced tomography (Xe/CT) offer a quantitative and regionally specific blood flow determination. However they are expensive and therefore not widely accessible. Diffusion imaging with magnetic resonance imaging provides excellent anatomic imaging; however at this time MRI is unable to differentiate gradients of flow unless diffusible tracers of CBF such as fluorocarbons are combined with imaging. Most of these methods are not suitable for evaluation of endothelial vasomotor role in CBF.

In contrast to the above methods transcranial Doppler (TCD) is a relatively simple, noninvasive, and low-cost bedside test that is widely available. It offers a unique real time velocity measurement of intracranial vessels. Although a single velocity measurement is not tightly linked to CBF, the change of velocity induced by a physiologic challenge is related to a proportional CBF change.

### 1.2. Transcranial Doppler Ultrasonography

Transcranial Doppler sonography (TCD) enables measurement of the blood flow velocity through the cerebral blood vessels. The method was introduced in 1982 by Aaslid and colleagues [15]. The value obtained for a particular artery is the velocity of blood flowing through the vessel, and unless the diameter of that vessel is established by some other means it is not possible to determine the actual blood flow. Thus TCD is primarily a technique for measuring relative changes in flow. The clinical utility of the technique is now well established for a number of different disease processes such as intracranial stenosis, evaluation of collateral blood flow, vasospasm in subarachnoid hemorrhage, and brain death [16].

Frequencies around 2 MHz are used for better penetration of ultrasound waves through the bone. However, the bones of the skull still block the transmission of ultrasound and regions with thinner walls—insonation windows—must be used for analyzing. For this reason, recording is performed in the temporal region above zygomatic arch (transtemporal insonation window), through the eyes (transorbital insonation window), below the jaw, and from the back of the head (transoccipital insonation window). Patient age, gender, race, and other factors affect bone thickness, making some examinations more difficult or even impossible. Most can still be performed to obtain acceptable responses, sometimes requiring using alternate sites from which the vessels are viewed

TCD does not include B-mode and is therefore a blind method. Identification of large cerebral vessels on the base of the brain is possible only by experienced ultrasonographer. It is based upon the direction of the flow as well as depth and angle of the insonation.

Functional transcranial Doppler sonography (fTCD) is a neuroimaging tool for measuring cerebral blood flow velocity changes due to neural activation during various tasks. Functional TCD uses pulse-wave Doppler technology to record blood flow velocities in the anterior, middle, and posterior cerebral arteries. Similar to other neuroimaging techniques such as functional magnetic resonance imaging or positron emission tomography, fTCD is based on a close coupling between regional cerebral blood flow changes and neural activation. Due to a continuous monitoring of blood flow velocity, TCD offers an excellent temporal resolution in comparison to other neuroimaging techniques. The technique is noninvasive and easy to apply. Blood flow velocity measurements are robust against movement artifacts. Since its introduction the technique has contributed substantially to the elucidation of the hemispheric organization of cognitive, motor, and sensory functions. fTCD has been particularly useful for the study of cerebral lateralization of major brain functions such as language, facial processing, color processing, and so forth [18].

Overall fTCD is a very sensitive tool but requires some knowledge for the interpretation of the results. The changes of blood flow velocity reflect blood flow changes only in certain conditions. The position of the probe has to be in the same spot during the whole recording in order to avoid changes due to the vessel diameter. In addition hyper- or hypoventilation strongly affects the blood velocity and blood flow. Therefore, CO_2_ concentration has to be monitored during all recordings and has to remain unchanged. In most recordings mean arterial pressure is also monitored since blood flow velocity reflects blood flow changes only in the autoregulation plateau.

### 1.3. L-Arginine

L-Arginine is in certain conditions an essential amino acid taking part in many biochemical reactions, for example, synthesis of NO. The most important role of L-arginine is its influence on vascular endothelial cells and hence on blood flow. It is well known that intravenous administration of L-arginine causes vasodilatation and augmented blood flow in many vascular beds [19–21]. Even more, L-arginine has been shown to play a role in lowering thrombotic activity, cell proliferation, inflammation, and other processes that lead to cardiovascular disorders. L-Arginine is involved in the prevention of atherosclerosis and effectively reduces the burden of atherosclerotic changes already present [22]. Thereby, it slows adhesion of monocytes to endothelium, lowers blood pressure in some hypertensive patients [22, 23], and brings endothelial function back to normal in patients with hypercholesterolemia [24]. The use of L-arginine has been proven to be safe [25].

So far, many studies have been published regarding the role of L-arginine in regulation of cerebral vasculature, predominantly in animal models. L-Arginine liberates NO by the catalytic reaction of NOS. Nitric oxide is known as the most effective vasodilator and as such the basic predictor of vascular tone, especially so in the cerebrovascular system [26]. Three isomorphic forms of NOS exist, namely, endothelial (eNOS), neuronal (nNOS), and inducible (iNOS). There is some data that eNOS is the cornerstone of NO-mediated vasodilatation of cerebral arteries and arterioles (Table 1) [17].

Nitric oxide is synthesized endogenously and exogenously. L-Arginine is the most important endogenous source of NO. In contrast to endogenous L-arginine, exogenous L-arginine liberates NO also by liberating other vasoactive substances [27, 28]. The crucial exogenous source of NO is the NO donors. They emit NO by mechanisms independent of NOS. Examples of such substances are organic nitrates (glyceryl trinitrate), sodium nitroprusside, and others.

**Figure 1:**
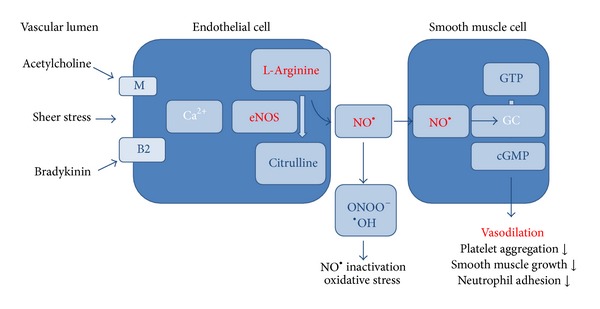
NO synthesis and its role.

Endothelium-derived NO is constantly released in baseline quantities, but different stimuli dynamically augment its formation. After being released, NO passes to nearby vascular smooth muscle and other cells. Its action is short-lived due to quick inactivation. In the target cells, NO activates guanylate cyclase (GC) and thence intracellular concentration of cyclic guanosine monophosphate (cGMP) rises. Elevated level of cGMP leads to reduced intracellular concentration of calcium (Ca^2+^) in the vascular smooth muscle cells with resultant vasodilatation (Figure 1).

Recently, there has been an increasing awareness about the dysfunction of cerebral endothelium playing an important role in various vascular disorders. It is known that dysfunction of cerebral endothelium can be brought about by various risk factors for cerebrovascular disease, metabolic disorders, and systemic and local inflammation.

## 2. Cerebrovascular Reactivity to L-Arginine

### 2.1. Physiological Conditions

Elevation of blood flow after L-arginine infusion has been discovered in animal models as well as in humans [29, 30]. The normal response to L-arginine is increased blood flow velocity as assessed by TCD [3–7] This probably reflects elevated blood flow through cerebral vasculature. However, the exact mechanism of L-arginine action on blood flow velocity in cerebral arteries remains incompletely elucidated. The method is based on noninvasive monitoring of blood flow velocity most commonly in the middle cerebral artery by means of TCD. According to the equation, cerebral blood flow (CBF) = *v*_*m*_ × cross-sectional area of the vessel. A potential limitation of this technique is that theincrease in *v*_*m*_ during L-arginine infusion could be either due to an increase in cerebral blood flow or constriction of the insonated artery. However, MCA constriction is very unlikely since it is well known that topical L-arginine induces vasodilatation [29]. The increase in *v*_*m*_ during L-arginine infusion is therefore probably due to an increase in CBF. As the CO_2_ concentration strongly influences CBF, it is very important to monitor it carefully. In most patients it does not change and therefore does not influence the overall *v*_*m*_ increase during L-arginine infusion. Cerebrovascular reactivity to L-arginine therefore probably reflects an increase in CBF and dilatation of the small vessels due to NO release.

Endothelial function is highly specific and can differ even within a vascular bed of a single organ. Cerebral endothelial function could therefore be different in distinct cerebrovascular territories. Furthermore, certain territories could be more susceptible to ischemia and stroke. Higher incidence and prevalence of stroke in males suggest that gender could have a strong influence on this difference. For this reason CVR-L-arg was compared in the anterior and posterior cerebral circulation in healthy young males and females. CVR-L-arg was significantly higher in the posterior cerebral artery (PCA) than in the middle cerebral artery (MCA). In addition, CVR-L-arg was significantly more pronounced in females compared to males in PCA and MCA. Lower CVR-L-arg and therefore lower cerebral endothelial function in the anterior cerebral circulation and in males could help to explain the higher incidence of ischemia and stroke in the anterior cerebral circulation, particularly in males [31].

### 2.2. Pathological Conditions

It is well known that endothelial function is impaired early in the course of atherosclerosis before morphological changes take place. Studies using flow-mediated dilatation (FMD) have shown that patients with arterial hypertension, diabetes, and other vascular risk factors have impaired systemic endothelial function [32–34]. CVR-L-arg has been used in several studies to determine cerebral endothelial function in these patients. A diminished cerebral endothelial function has been shown in patients with hypertension [35], diabetes mellitus [36], and patients with lacunar infarctions [35]. CVR-L-arg in the patients with lacunar infarctions was significantly lower compared to the healthy controls but similar to that obtained in the patients with comparable cardiovascular risk factors [35]. Since endothelial dysfunction of the noncerebral arteries in patients with cerebrovascular risk factors has been proven many times [30, 32] the diminished CVR-L-arg in the two patient groups was expected. However, the studies of endothelial dysfunction in stroke patients provide contradicting results, with some authors reporting a decrease [4, 6], whereas others found an increase in CVR-L-arg [7]. Since several mechanisms are involved in the pathogenesis of stroke it is possible that patient selection is crucial to the heterogeneity of the results. Studies included patients with large middle cerebral artery infarctions [6], patients with symptomatic internal carotid artery stenosis [4], and a rather undefined group of patients with smaller infarctions and TIAs [7]. In a study where a homogeneous group of patients with lacunar infarctions was included a clear decrease of CVR-L-arg was shown.

Cardiovascular risk factors such as hypertension and hypercholesterolaemia suggest that patients with lacunar infarctions have reduced synthesis and/or increased oxidative breakdown of NO [32]. However, these observations refer only to the endothelium of noncerebral arteries. Only recently researchers have clarified the role of NO and different NOS isoforms in cerebral ischaemia [17]. In addition to a wide range of physiological protective roles NO has a paradoxical role in the acute ischemic conditions due to its formation of highly toxic free radicals which further damage ischemic tissue, hence the so called NO-mediated brain damage.

Animal studies have shown that adaptation to cerebral ischaemia leads to induced synthesis of all three NOS isoforms and an increase in NO production [36]. Neuronal nNOS plays a prominent role in the early stage (10 minutes) which is followed by endothelial eNOS (1 hour) and macrophageal inducible iNOS (12 hours) (Table 1). Once upregulated, iNOS produces large amounts of NO, which can damage or even destroy cells. The activity of the inducible iNOS peaks only after 2 days and stays active up to 7 days [37]. As a consequence the concentration of the serum L-arginine decreases after the ischemic event [38].

Infusion of L-arginine during the acute phase of stroke could therefore result in the production of free radicals and could be harmful. In fact NOS inhibitors have been shown to minimize the ischemic reperfusion injury after acute stroke [39, 40]. Further studies using pan-NOS inhibitors yielded conflicting results [41] most likely due to their differential activity on the three different NOS isoforms (eNOS: protective and nNOS/iNOS: destructive). However, outside the acute and subacute phase of stroke NOS isoforms are no longer induced. In fact, only nNOS and eNOS are constitutively expressed [17] and their activity is reflected by CVR to L-arg. By definition, patients with cerebral infarctions have definite necrotic areas of the brain and therefore a quantitative endothelial loss, which also means the loss of eNOS. In addition NO production is also influenced by all the risk factors. One of these mechanisms probably prevails in a subgroup of stroke patients and explains the conflicting results of CVR to L-arg described by different authors.

The method has also been used in order to determine the efficacy of therapy. Statin treatment significantly improved CVR-L-arg both in patients with lacunar infarctions and in patients with similar risk factors [42]. This finding could probably be explained by the beneficial effects of statins on endothelial function. Statins are known to activate eNOS and thereby propagate NO-dependant vasodilatation. eNOS conformation changes and NO production decreases under the influence of almost all cerebrovascular risk factors [43]. However, animal studies have shown that adaptation to cerebral ischaemia leads to eNOS upregulation [36] and an increase in NO production [17, 44]. Another aspect of this problem is the presence of definite necrotic areas of the brain and therefore a quantitative endothelial loss, which also means loss of eNOS. CVR-L-arg probably reflects all these and possibly other effects. Since some factors downregulate and others upregulate the cerebral NO production in patients with lacunar infarctions it was not entirely surprising that the CVR-L-arg in patients with similar cerebrovascular risk factors was equally decreased. Nevertheless, the most important finding was that at least some of these mechanisms influencing eNOS could be reversed by atorvastatin therapy, not only in asymptomatic patients with cerebrovascular risk factors, but also in patients with lacunar infarctions. Importantly, the cholesterol values of patients with lacunar infarctions and asymptomatic patients with similar risk factors were only mildly elevated before atorvastatin therapy and normalised afterwards. The results were in agreement with previous studies, in which the improvement in endothelial function after statin therapy was attributed not only to the normalisation of the cholesterol values but also to other protective mechanisms [45–47]. Importantly, these findings suggested that statin therapy should be considered in the prevention of multi-infarct cerebrovascular disease. In addition to in vitro experiments, animal studies, and epidemiological data this was unique in vivo evidence that helped make statin therapy obligatory in the secondary prevention of noncardioembolic stroke [48].

CVR-L-arg has also been determined in patients with migraine. The role of endothelium in the pathophysiology of migraine patients is not well known. Cerebral infarction preferentially affects the posterior cerebral artery distribution in migraine patients. Migraine patients had lower CVR-L-arg in PCA and similar in MCA compared to healthy subjects. Lower CVR to L-arginine in PCA in migraine patients could help to explain the observation that stroke is much more frequent in the anterior than in the posterior circulation [49].

## 3. Cerebrovascular Reactivity to L-Arginine Compared to Other Methods

CVR-L-arg has been compared to other methods for evaluation of endothelial function and morphology. Flow-mediated dilatation (FMD) was used as a marker of systemic endothelial function and carotid intima-media thickness (IMT) as a well-known marker for endothelial morphological evaluation. In previous studies the combination of IMT and FMD has been frequently used to evaluate endothelial impairment [50, 51]. Correlations were analysed in a heterogeneous group consisting of patients with lacunar infarctions and extensively impaired endothelial function, patients with similar risk factors, but without lacunar infarctions, and healthy controls. Analysis of correlations revealed that CVR-L-arg did not correlate with FMD. On the contrary, a significant negative correlation was found between CVR-L-arg and IMT. The study showed that cerebral endothelial function, determined by L-arginine reactivity, correlates well with the degree of atherosclerosis determined by IMT but does not correlate with FMD, suggesting that cerebral and systemic endothelial function may not be closely associated. As previously thought this pointed out that CVR-L-arg enables detection of highly specific cerebral endothelial function [52].

Although the functional changes measured by FMD have been found to correlate well with the structural changes of the arteries measured by IMT [50, 51], it has been shown that FMD is already diminished in the early stages of atherosclerosis, whereas IMT is increased only in more advanced atherosclerosis [53]. We found that cerebrovascular reactivity to L-arginine correlated significantly with IMT, but not with FMD. This could be explained by the different nature of the endothelial parameters. FMD enables evaluation of the present endothelial function, while IMT reflects the cumulative effect of endothelial impairment.

It is well known that FMD is a functional method, based on arterial diameter changes that occur mainly as a result of endothelial release of NO that correlates with coronary endothelial function [54, 55]. In contrast, IMT of the common carotid arteries reflects morphological changes in the endothelium. However, IMT measurements reflect atherosclerosis as well as nonatherosclerotic intimal reactions that could be present in small vessel disease such as lacunar infarctions. Cerebrovascular reactivity to L-arginine probably reflects functional as well as morphological changes in the cerebral endothelium. Under the influence of almost all cardiovascular risk factors, the eNOS conformation changes and NO production is diminished [56, 57]. However, animal studies have shown that adaptation to cerebral ischaemia leads to eNOS upregulation [44] and an increase in NO production [45]. Brain necrosis with subsequent quantitative endothelial loss means loss of eNOS, which also influences L-arginine cerebrovascular reactivity. L-Arginine cerebrovascular reactivity therefore probably reflects the loss of endothelial tissue as well as several factors that influence eNOS. The three methods therefore probably reflect different aspects of endothelial disease and thus they cannot simply be compared. In the study it was concluded that cerebral endothelial function, determined by L-arginine reactivity, correlates well with the degree of atherosclerosis determined by IMT but does not correlate with FMD, suggesting that cerebral and systemic endothelial function may not be closely associated.

CVR-L-arg has also been compared to FMD and carotid intima-media thickness (IMT) in order to show the specific effects of statin therapy on cerebral endothelial dysfunction. Statin treatment has been shown to improve cerebral more than systemic endothelial function [58]. Cerebral and systemic endothelial functions were compared in patients with arterial hypertension (AH) and healthy controls before and after atorvastatin treatment. The measurements were repeated after a three-month treatment with atorvastatin. L-Arginine reactivity and FMD were decreased in patients with AH compared to controls. After atorvastatin treatment, L-arginine reactivity and FMD improved in patients with AH compared to controls. The L-arginine reactivity improved to a degree undistinguishable from controls, whereas FMD did not. Statin use restored the cerebral circulation reactivity, while there was little change in the systemic circulation measured by FMD. The decreased L-arginine reactivity and FMD were found to improve after atorvastatin treatment in patients with AH but the results suggest that statin therapy improved cerebral more than systemic endothelial function [58].

## 4. Methodology

**Figure 2:**
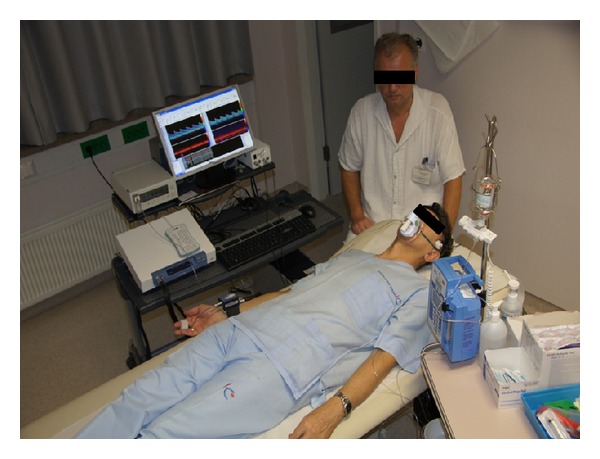
Cerebrovascular reactivity to L-arginine. The subject rests in a supine position. Two 2 MHz probes are immobilized by a handle on the subjects' head and *v*_*m*_ from both middle cerebral arteries (MCA) is recorded by TCD. Blood pressure and heart frequency on the right radial artery are monitored as well as CO_2 _concentration in the exhaled air. All signals appear on the same time scale on the computer. Intravenous infusion of L-arginine is injected into the left brachial vein by a pump.

Due to the diurnal variations the measurement should take place at the same time of the day preferably in the morning and the participants should be fasted. The measurements should take place in a calm room with constant temperature. The subject is advised to first lie down for at least 15 minutes in order to rest and relax. By using TCD with two 2 MHz probes a signal from both middle cerebral arteries (MCA) at the depth of about 50–54 mm through the temporal acoustic windows should be found and the probes should then be immobilized by the means of a special handle (Figure 2).

At the same time measurement of blood pressure and heart frequency on the left radial artery and CO_2_ concentration in the exhaled air by the means of ventilation mask connected to capnometer should be performed. In relatively rare cases a patient cannot tolerate breathing through a mask and starts hyperventilating or falls asleep and periods of hypopnea and apnea with retention of CO_2_ appear. In case of a significant CO_2_ change the recording is of no use.

All the signals are preferably gathered on a single computer so that simultaneous assessment of all the parameters is possible. In case this is not possible time markers at the beginning and at the end of stimulation for each parameter are obligatory for later analysis.

An intravenous line should be placed into the right cubital vein. In case blood is needed for laboratory analysis it can be withdrawn before the infusion of L-arginine. By the action of a pump a 30-minute lasting infusion of 100 mL of 30-percent L-arginine chloride (L-arginine HCL) through intravenous line should be administered. All the measurements should take place when the participant is at rest (15 minutes), during the 30-minute infusion of L-arginine, and another 20 minutes after the cessation of infusion.

TCD software is used to determine *v*_*m*_ during the 10-minute rest interval and the 10-minute interval after L-arginine infusion. *v*_*m*_ should be calculated according to the formula:

(1) $$\begin{array}{c} v_{m}=\int \frac{vdt}{t_{0}-t_{10}}, \end{array}$$

where *v*_*m*_ is the mean blood flow velocity in MCA. CVR to L-arginine in MCA is calculated according to the following formula:

(2) $$\begin{array}{c} \Delta v_{\text{L-arg}}=\frac{v_{m}\left(\text{during}\;\text{L-arginine}\;\text{infusion}\right)-\;v_{m}\left(\text{at}\;\text{rest}\right)}{v_{m}\left(\text{at}\;\text{rest}\right)}. \end{array}$$

MAP, heart rate, CO_2,_ and Et-CO_2_ have to be calculated for the same intervals as *v*_*m*_ preferably using TCD software. The paired *t-*test should be used to compare *v*_*m*_, MAP, heart rate, CO_2,_ and Et-CO_2_ before and after intravenous infusion of L-arginine

## 5. Conclusion

Cerebrovascular reactivity to L-arginine is a unique inexpensive method that enables a noninvasive evaluation of cerebral endothelial function. Following a strict standardized protocol the method enables reproducible measurements. Its diagnostic utility for evaluation of endothelial impairment has been compared to flow-mediated dilatation as a gold standard for systemic endothelial function and intima-media thickness as a marker for morphological changes. However, it seems to show specific cerebral endothelial function. Cerebrovascular reactivity to L-arginine is a promising noninvasive research method that could provide means for evaluation of cerebral endothelial function in physiological and pathological conditions.

## Figures and Tables

**Table 1:** Types of nitric oxide synthase and their role in cerebral ischaemia [17].

nNOS (NOS I)	iNOS (NOS II)	eNOS (NOS III)
Activity depends on elevated Ca^2+^	Activity is independent of Ca^2+^	Activity depends on elevated Ca^2+^
First identified in neurons	First identified in macrophages	First identified in endothelial cells
Constitutively expressed, but inducible under pathological conditions	Inducible under pathological conditions	Constitutively expressed, but inducible under pathological conditions
Plays a prominent role in the early stage of neuronal injury after cerebral ischemia	Plays a role in the later stages of neuronal injury after cerebral ischemia	Plays a protective role in cerebral ischemia by maintaining cerebral blood flow
Protein and catalytic activity upregulated within 10 minutes and peak at 3 hours after cerebral ischemia	Protein and catalytic activity upregulated within 12 hours and peak at 48 hours after cerebral ischemia	Protein and catalytic activity upregulated within 1 hour and peak at 24 hours after cerebral ischemia
